# Bone mesenchymal stem cells are recruited via CXCL8‐CXCR2 and promote EMT through TGF‐β signal pathways in oral squamous carcinoma

**DOI:** 10.1111/cpr.12859

**Published:** 2020-06-26

**Authors:** Lin Meng, Yueqi Zhao, Wenhuan Bu, Xing Li, Xinchen Liu, Dabo Zhou, Yumeng Chen, Shize Zheng, Quan Lin, Qilin Liu, Hongchen Sun

**Affiliations:** ^1^ Department of Oral Pathology Jilin Provincial Key Laboratory of Tooth Development and Bone Remodeling School and Hospital of Stomatology Jilin University Changchun China; ^2^ State Key Laboratory of Supramolecular Structure and Material College of Chemistry Jilin University Changchun China; ^3^ School and Hospital of Stomatology China Medical University Shenyang China

**Keywords:** bone mesenchymal stem cells, chemotaxis, CXCL8, epithelial‐mesenchymal transition, oral squamous cell carcinoma, TGF‐β/Ras/Raf/Erk

## Abstract

**Objectives:**

Bone mesenchymal stem cells (BMSCs) play critical roles in tumour microenvironment. However, molecular mechanisms of how BMSCs to be recruited and effect subsequent tumour progression are poorly understood in oral squamous cell carcinoma (OSCC).

**Materials and Methods:**

The distribution of CXCL8 was detected by immunohistochemical staining in OSCC tissues. The chemotaxis of conditioned media from different epithelial cells to BMSCs was examined by trans‐well assay. Real‐time quantitative PCR (qPCR) and ELISA were used to detect the expression of related cytokines and chemokine receptors. The migration of BMSCs was observed in BALB/c nude mice. The roles of BMSCs in proliferation, migration and invasion of OSCC were detected by CCK‐8, flow cytometry and trans‐well assay. Epithelial‐mesenchymal transition (EMT)–related markers were analysed by qPCR and Western blot in vitro, and growth was evaluated in BALB/c nude mice using subcutaneously implanted OSCC in nude mouse model in vivo.

**Results:**

Using OSCC, we show CXCL8, secreted by OSCC, binds to exclusively CXCR2 in BMSCs to facilitate migration of BMSCs to OSCC. TGF‐β secreted by BMSCs subsequently induces EMT of OSCC to promote their proliferation, migration and infiltration. We also showed that the Ras/Raf/Erk axis plays a critical role in tumour progression.

**Conclusions:**

Our results provide the molecular basis for BMSC recruitment into tumours, and how this process leads to tumour progression and leads us to develop a novel OSCC treatment target.

## 1 INTRODUCTION

Oral squamous cell carcinoma (OSCC) is the most common malignant tumour in head and neck squamous cell carcinoma (HNSCC).^1^ Furthermore, HNSCC still lacks optimized treatment, and there are still about 50% of HNSCC patients with localized regional recurrence and metastasis (especially tongue squamous cell carcinoma and oropharyngeal squamous cell carcinoma).^1^, ^2^ Therefore, to improve OSCC’s treatment, we still need to reveal deep molecular mechanisms of OSCC proliferation leading to develop new effective treatment.

BMSCs become an especially important tool for regenerative medicine and a major cellular source for the biological therapies because of their self‐renewal capacity, multipotency and immunomodulatory properties. It has been reported that BMSCs can be recruited into tumour microenvironment (TME) in response to a variety of biologically active molecules to repair tissues, balance cell homeostasis and participate in immune regulation.^3^ BMSCs can be recruited by all kinds of chemokines in various cancers, for example CXCL16 in prostate cancer,^4^ CXCL12 in breast cancer,^5^ CXCL8/IL‐8, CCL2/MCP‐1 and CXCL1‐2‐3/GRO that play a role in the homing of BMSCs to liver tumour sites,^6^ CCL5,^7^ TGF‐β and interleukin‐17b,^8^ CXCL10, placental growth factor ^9^ and platelet‐derived growth factor receptor beta.^10^ However, there is no report to uncover the chemotaxis mechanism of BMSCs in OSCC.

In cancers, however, BMSCs can secrete multiple bioactive factors to alter the key cellular functions of cells in TME, such as survival, apoptosis, maturation and differentiation.^11^, ^12^, ^13^ On the one hand, BMSCs play a vital role in breast cancer development.^14^ BMSCs promote the spread of breast cancer cells.^15^ In osteosarcoma, exosomes secreted by BMSCs promote osteosarcoma cell proliferation.^16^ On the other hand, in malignant gliomas, BMSCs can interact with TME, which leads to tumour shrinkage, and impair cell proliferation, vascularization and significantly prolonged animal survival.^17^ BMSCs prevent the development of oncogenic lung cancer in the rat model.^18^ In addition, it was also found that BMSCs have different effects on different biological behaviours of the same type of tumour. In thyroid cancer, BMSCs promote cancer cell proliferation and block cancer cell migration.^19^ All these suggest that changes in TME affect the differentiation and proliferation of cancer cells. In this study, we tried to explore the chemotaxis of BMSCs and the role of BMSCs in OSCC migration, invasion and EMT by using in vitro and in vivo experiments.

## 2 MATERIALS AND METHODS

### 2.1 Patient samples and clinicopathological data

All patients' samples used in this study were performed in accordance with the Declaration of Helsinki. Collections and use of these samples were approved by the ethical review committees of the Jilin University Stomatology Hospital. Human tumour tissues, epithelial dysplasia and normal adjacent tissues were obtained from 30 subjects who came for surgery treatment of OSCC (16 males and 14 females) at the Jilin University Stomatology Hospital. All subjects did not have any treatment before surgery. Median age was 56 years (range 22‐81). There were 6 gingival tumours, 13 tongue tumours, 4 buccal tumours, 3 oral floor carcinoma, 2 oropharyngeal cancer and 2 lip carcinoma. There were 8 samples at high grade of OSCC, 10 at intermediate grade of OSCC, 10 at low grade of OSCC, 10 from epithelial dysplasia and 10 from normal adjacent tissues (Table 1). Tumour tissues and relatively normal mucosal tissues were fixed by 10% formalin and embedded in paraffin. The study was approved by the ethics committee of Jilin University Stomatology Hospital.

**Table 1:** Detailed clinical information for each patient whom clinical sample was obtained from

Patients	Gender	Age (y)	Diagnosis	Pathological grades	Location	Lymphatic metastasis
1	male	42	OSCC	intermediate	Mouth floor	no
2	female	62	OSCC	intermediate	Tongue	no
3	male	51	OSCC	low	Buccal	no
4	female	70	OSCC	high	Tongue	no
5	male	56	OSCC	low	Mouth floor	no
6	male	68	OSCC	low	Maxillary sinus	no
7	female	60	OSCC	high	Tongue	no
8	female	50	OSCC	high	Tongue	no
9	female	73	OSCC	high	Gingival	no
10	female	69	OSCC	high	Lower lip	no
11	male	38	OSCC	high	Tongue	no
12	male	73	OSCC	low	Tongue	no
13	male	45	OSCC	low	Oropharyngeal	no
14	male	54	OSCC	intermediate	Tongue	no
15	male	79	OSCC	low	Gingival	no
16	female	22	dysplasia	‐	Gingival	no
17	male	49	OSCC	intermediate	Mouth floor	no
18	male	38	dysplasia	‐	Buccal	no
19	male	64	OSCC	intermediate	Oropharyngeal	no
20	male	63	OSCC	low	Gingival	Yes
21	female	81	OSCC	high	Tongue	no
22	male	70	OSCC	intermediate	Gingival	no
23	female	52	OSCC	intermediate	Buccal	no
24	female	50	OSCC	low	Buccal	no
25	male	42	OSCC	intermediate	Tongue	no
26	female	72	OSCC	high	Tongue	no
27	male	41	OSCC	intermediate	Gingival	no
28	female	51	OSCC	low	Gingival	no
29	female	44	OSCC	intermediate	Tongue	no
30	male	51	OSCC	low	Tongue	Yes

### 2.2 Primary culture of BMSCs

Human BMSCs were isolated from the bone marrow of patients who undergone mandibular reconstruction with free fibular flap. Briefly, bone marrows were centrifuged by density gradient centrifugation with 1.073 g/mL Percoll (Sigma‐Aldrich) at 1810 *g* for 30 minutes, and BMSC layer was collected and cultured in DMEM supplemented with 20% FBS, 100 U/mL penicillin and 100 μg/mL streptomycin. Then, BMSCs from passage 3 were further analysed and confirmed using CD90 (Biolegend, San Diego, CA, USA), CD105 (Biolegend) and CD44 (Biolegend) for positive surface marks and CD11b (Biolegend) and CD45 (Biolegend) for negative surface marks by a FACSCanto flow cytometer. Experiments were performed using BMSCs between passages 3 and 6 (Figure S3).

### 2.3 In vivo migration assay of BMSCs

All animals used in this study were approved by the Institutional Animal Care and Use Committee of Jilin University and maintained under specific pathogen‐free conditions. All animal procedures were conducted according to the guidelines approved by the China Association of Laboratory Animal Care.

To further evaluate the migration of BMSCs in vivo, BMSCs were marked with 40μg/ml Au‐PEI. Nine male BALB/c‐nu/nu mice (4‐6 weeks old) (Vital River Laboratory Animal Technology, Beijing, China) were used to create subcutaneous tumours by injecting CAL27 cells (2 × 10^6^ cells/mouse) into the dorsal flank. After tumour diameters reached about 5 mm on 3 weeks, mice were randomly divided into three groups (n = 3), BMSCs (at 1 × 10^6^ cells)‐Au‐PEI, BMSCs (at 1 × 10^6^ cells)‐Au‐PEI‐SB225002 and Au‐PEI groups. Then, these three different combinations were injected into different mice through the tail vein. Mouse was anaesthetized by inhalation of a mixture of oxygen with 5% sevoflurane, photographed under IVIS (In Vivo Imaging Instruments, KODAK) using spectrum imaging system at Ex/Em 420 nm/600 nm after 24 hours post‐injection. Then, heart, liver, spleen, lung, kidney and tumour were collected, rinsed in saline and photographed. Tumours were further frozen‐sectioned, dying the nucleus with DAPI. Distribution of BMSCs‐Au‐PEI was assessed by direct visualization using fluorescence microscope.

### 2.4 Effect of BMSCs on OSCC in vivo

Twenty‐five male BALB/c nude mice (6 weeks old, 20‐25 g) were randomly divided into five groups (n = 5), CAL27, BMSCs + CAL27, co‐CAL27, co‐CAL27 + U0126 and co‐CAL27 + SB431542. CAL27 cells were treated with U0126 or SB431542 (10 nM/ml) for 24 h prior to co‐culture with BMSC‐CM, and then co‐cultured with BMSC‐CM + U0126 or SB431542 (10 nM/mL) for 5 days prior to in vivo injection. Different treated 2 × 10^6^ cells of CAL27 were injected into the dorsal flank to establish subcutaneous tumours. The size of the tumour was measured every 3 days until 39 days after 7 days of injection. Tumour volume was calculated with the formula a × b^2^ × 0.5, where a is the largest diameter, and b is the smallest diameter. After 40 days, tumour xenografts were collected, fixed, paraffin‐embedded, sectioned and stained with H&E and IHC detection by Ki67, vimentin and snail. The same experiment was repeated once. Therefore, a total of 50 male BALB/c nude mice were used. Some mice were lost during the experiments. At the last time point, 6 mice left for CAL27 group, 8 mice for BMSCs + CAL27, 6 for co‐CAL27, 4 for co‐CAL27 + U0126 and 4 for co‐CAL27 + SB431542 from both experiments. Tumorigenesis occurred in all mice used in this experiment. Figure 6A shows the results obtained from both the experiments, and Figure 6B shows the results obtained from second in vivo experiment.

### 2.5 Statistical analyses

All data were analysed using SPSS 19.0 statistical software. One‐way ANOVA was used to compare means of two groups or more. All tests were two‐sided. Data were presented as mean ± SD. All experiments in vitro were repeated three times. *P* values <.05 were considered to be significant.

## 3 RESULTS

### 3.1 CXCL8 and CXCL8 receptor expression profiles in BMSCs in vitro

**Figure 1:**
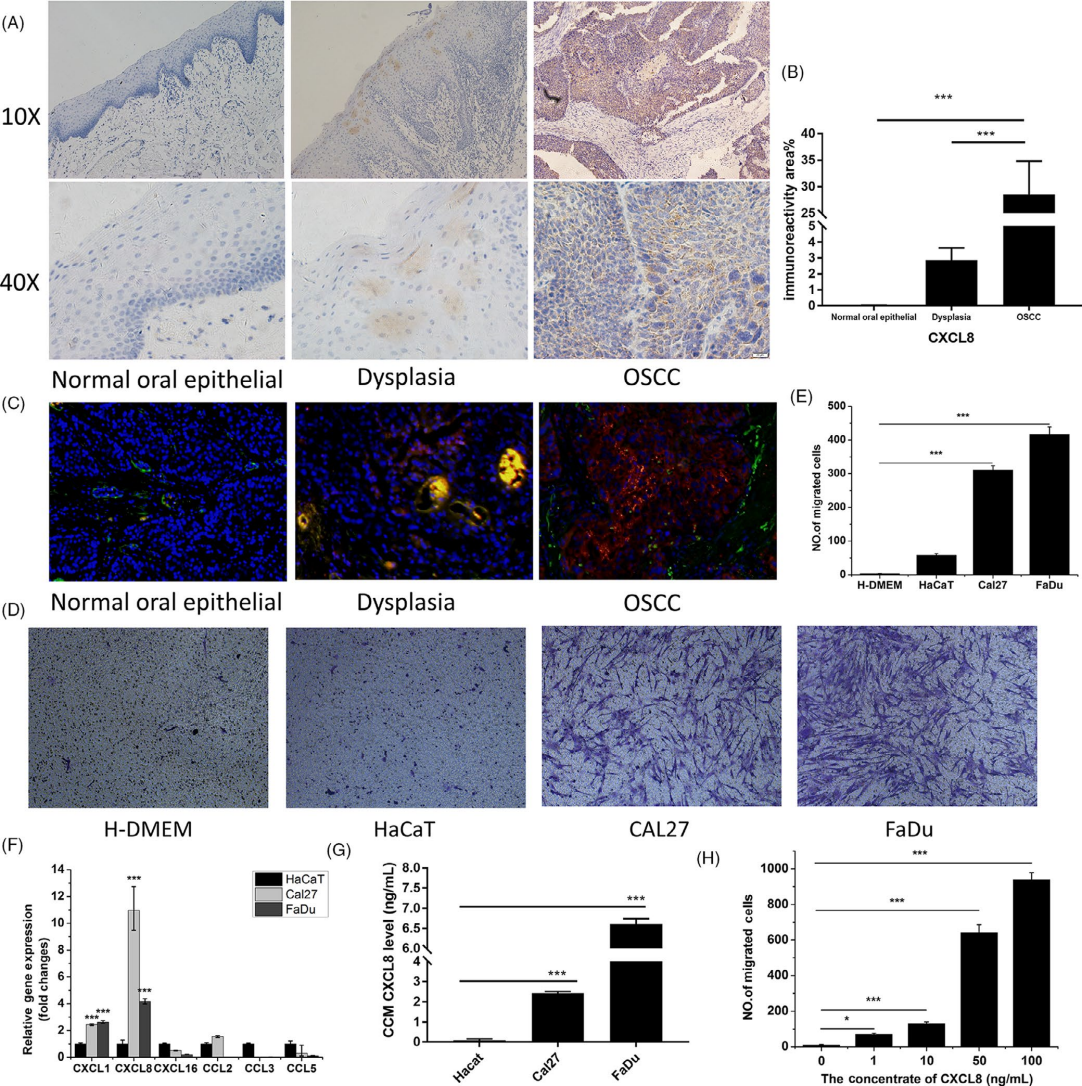
Expression of CXCL8 in OSCC, OSCC cell‐produced CXCL8 mediates the migration of BMSCs in vitro. A, Representative CXCL8 immunohistochemical staining for normal oral epithelial, dysplasia and human OSCC tissues. (10× and 40×) B, Immunoreactivity score indicating CXCL8 expression levels for normal oral epithelial (n = 10), dysplasia (n = 10) and OSCC cells (n = 10). C, Red fluorescence was positive CXCL8 staining, and green fluorescence was positive CD105 BMSC staining in clinical samples. Blue fluorescence was DAPI nuclear staining (20×). D, Representative photographs of stained filters showing migrated BMSCs. The number of cells that had migrated to the lower side of the filter was counted under a light microscope with five fields (magnification, ×100). E, Migration of BMSCs towards cell‐conditioned media (CCM) from OSCC cell lines, CCM from a normal epithelial cell line and H‐DMEM. F, The differentially relative mRNA expressed between OSCC cell lines and HaCaT cells. (G) CXCL8 levels in CCM from CAL27, FaDu and HaCaT cells. The concentrations of CXCL8 were measured by enzyme‐linked immunosorbent assay. (H) Recombinant CXCL8 stimulates the migration of BMSCs. **, *P* < .01; and ***, *P* < .001

Data from immunochemistry staining demonstrated that expression of CXCL8 was the highest in OSCC, second is oral epithelial dysplasia expression, and expression of CXCL8 was hardly detected in normal oral epithelial cells (Figure ). There were significant differences between OSCC and oral epithelial dysplasia or normal oral epithelial cells (Figure 1B). CXCL8 was mainly expressed in abnormal hyperplasia epithelium and carcinoma nests, and was rarely seen in tumour stroma. To evaluate relation between CXCL8 and BMSCs using clinical samples, double immunofluorescence staining was used. Data showed that there was almost no CXCL8 secretion with fewer BMSCs (CD105‐positive cells) in the normal oral epithelial cells, and there were a little more CXCL8 secretion and BMSCs around blood vessels (co‐localization shown in orange colour) in the epithelial dysplasia (Figure 1C). However, there were much more positive CXCL8 secretion and BMSCs in the OSCC (Figure 1C). These results indicate that BMSCs and OSCC have closer relation to CXCL8.To mimic in vivo migration of BMSCs, trans‐well assays were performed. Data showed that CCMs from CAL27 and FaDu significantly induced BMSCs cross the membrane compared with CCM of HaCaT and H‐DMEM medium control (Figure 1D and 1E). CCM from FaDu was the strongest one, CCM from HaCaT only had ~ 70 cells cross the membrane, and H‐DMEM medium control had no cell cross (Figure 1D and 1E). To confirm what component in the CCM played an important role in the BMSC migration, gene expression profiles for several cytokines were checked. Data from Figure 1F demonstrated that expression of CXCL8 was stronger in CAL27 and FaDu cell lines, and the strongest in CAL27 cell line compared with HaCaT cell line. Furthermore, ELISA results found out that there were much higher levels of CXCL8 protein in CCMs from CAL27 and FaDu, CCM from FaDu had the highest level, and CCM from HaCaT had no CXCL8 (Figure 1G). Interestingly, we recognized that migration of BMSCs was dose‐dependent on CXCL8, migration of BMSCs increases with the increase in CXCL8 (Figure 1H). These data indicate that CXCL8 plays critical role in the migration of BMSCs in vitro. In situ staining from Figure 1A also shows that the CXCL8 is an interesting cytokine (Figure 1A). CXCR1 and CXCR2 are cognate receptors for CXCL8. Data from co‐cultures demonstrated that OSCC cell lines, CAL27 and FaDu, dramatically induced gene expression of CXCR2 in BMSCs after BMSCs co‐cultured with CAL27 (Figure S1A and S1B). Importantly, SB225002 inhibitor, which inhibits CXCL8 binding to CXCR2, could effectively and significantly block BMSC migration in trans‐well assay (Figure S1C and S1D). These further confirm that CXCL8 plays critical role through CXCL8‐CXCR2 interaction in the migration of BMSCs.

### 3.2 Effects of CXCL8‐CXCR2 on BMSC migration in vivo

**Figure 2:**
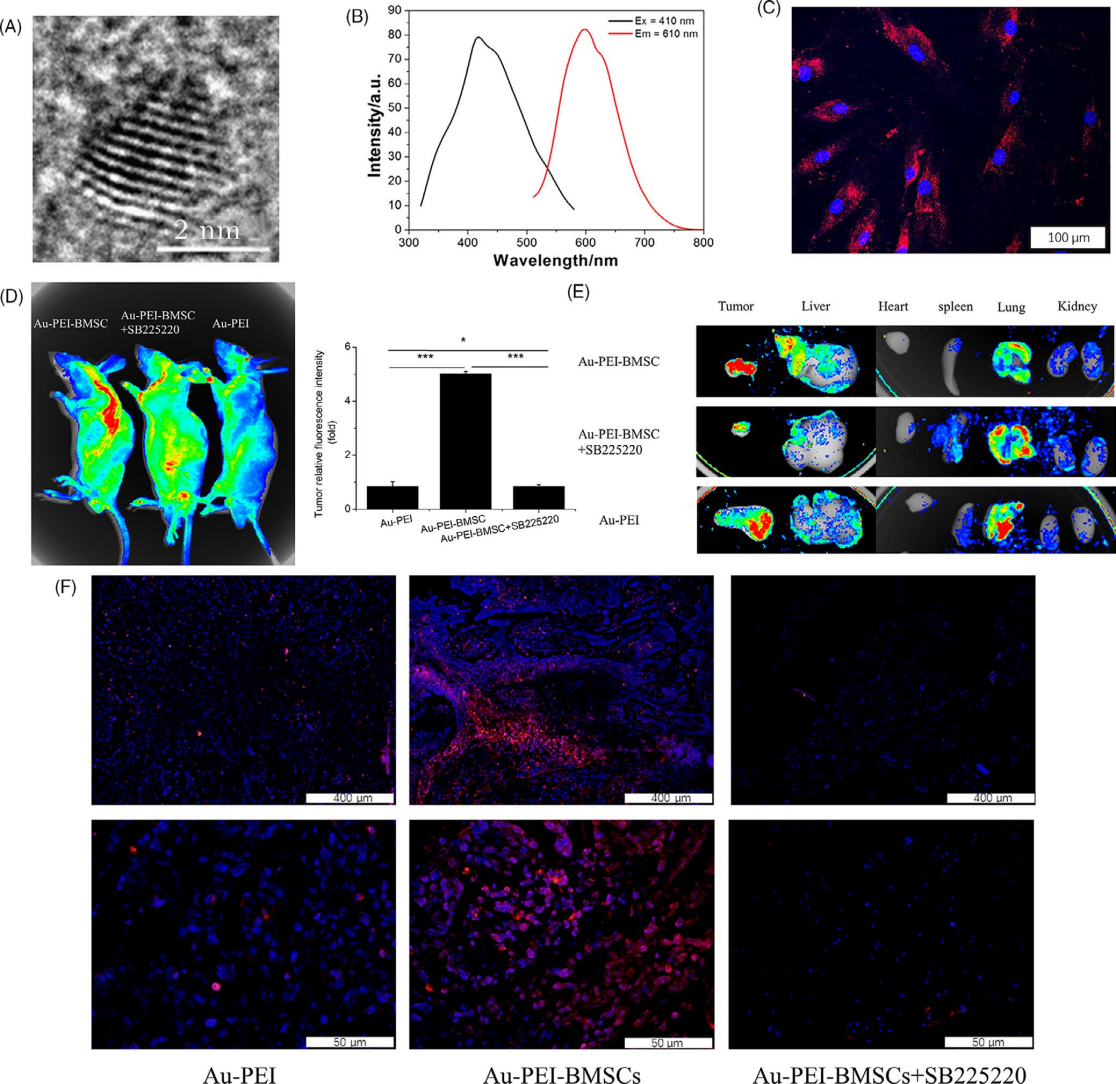
CXCL8‐CXCR2 mediates the BMSCs to OSCC tumour microenvironment in vivo. A, HRTEM image of Au‐PEI. The inset shows the size distribution of Au‐PEI. B, Fluorescence excitation and emission spectra of Au‐PEI. C, Laser confocal fluorescence micrographs of Au‐PEI. D, IVIS images of mice injected with Au‐PEI‐BMSCs, Au‐PEI‐BMSCs + SB225002 and Au‐PEI after 24 h. Tumour relative fluorescence intensity was shown in histogram. E, Tumour, liver, heart, spleen, lung and kidney excised from the above‐mentioned mice. F, Representative fluorescence of tumour sections from the above‐mentioned mice. Au‐PEI‐BMSCs were red. Nuclei were stained with DAPI. Scale bar = 400 μm (upper panels), Scale bar = 50 μm (lower panels). *, *P* < .05; **, *P* < .01; and ***, *P* < .001

To evaluate whether CXCL8‐CXCR2 plays the same role as in vitro in BMSC migration towards OSCC in vivo, CAL27 cell lines were used to establish xenograft animal model of OSCC. In order to track migration of BMSCs, BMSCs were labelled by Au‐PEI. Figure 2A and 2B shows that Au‐PEI synthesized for this study was a 2‐3 nm with red fluorescence, which could efficiently label BMSCs (Figure 2C). After 24‐h delivery, mice were detected by fluorescence molecular tomography. Figure 2D clearly shows that there were few small red fluorescence spots in Au‐PEI group, was very stronger and big red fluorescence spot, which was located at the xenograft tumour area in the Au‐PEI‐BMSC group, and was significant and dramatical decrease in red fluorescence in Au‐PEI‐BMSCs + SB225002 compared with the Au‐PEI‐BMSC group, there were significant differences between Au‐PEI‐BMSC group and Au‐PEI group or Au‐PEI‐BMSCs + SB225002. In order to exclude possible interference of autofluorescence, tumour, liver, heart, spleen, lung and kidney were isolated and collected to observe them in the dissociated situation. Figure 2E shows that red fluorescence signals were mainly in the tumour and very few in the liver and lung in the Au‐PEI‐BMSC group, while red fluorescence signals were significantly decreased and mainly in the lung and very few in the tumour in the Au‐PEI‐BMSC + SB225002 group. Compared with these two groups, red fluorescence signals were observed in the lung and some tumours were observed in the Au‐PEI group (Figure 2E). As shown (Figure 2F), BMSCs migrated to tumour stroma in OSCC rather than tumour. These in vivo experimental data, especially data from SB225002 inhibitor, demonstrate that CXCL8/CXCR2 also play an important role in the migration of BMSCs.

### 3.3 Effects of BMSCs on proliferation and apoptosis of OSCC

**Figure 3:**
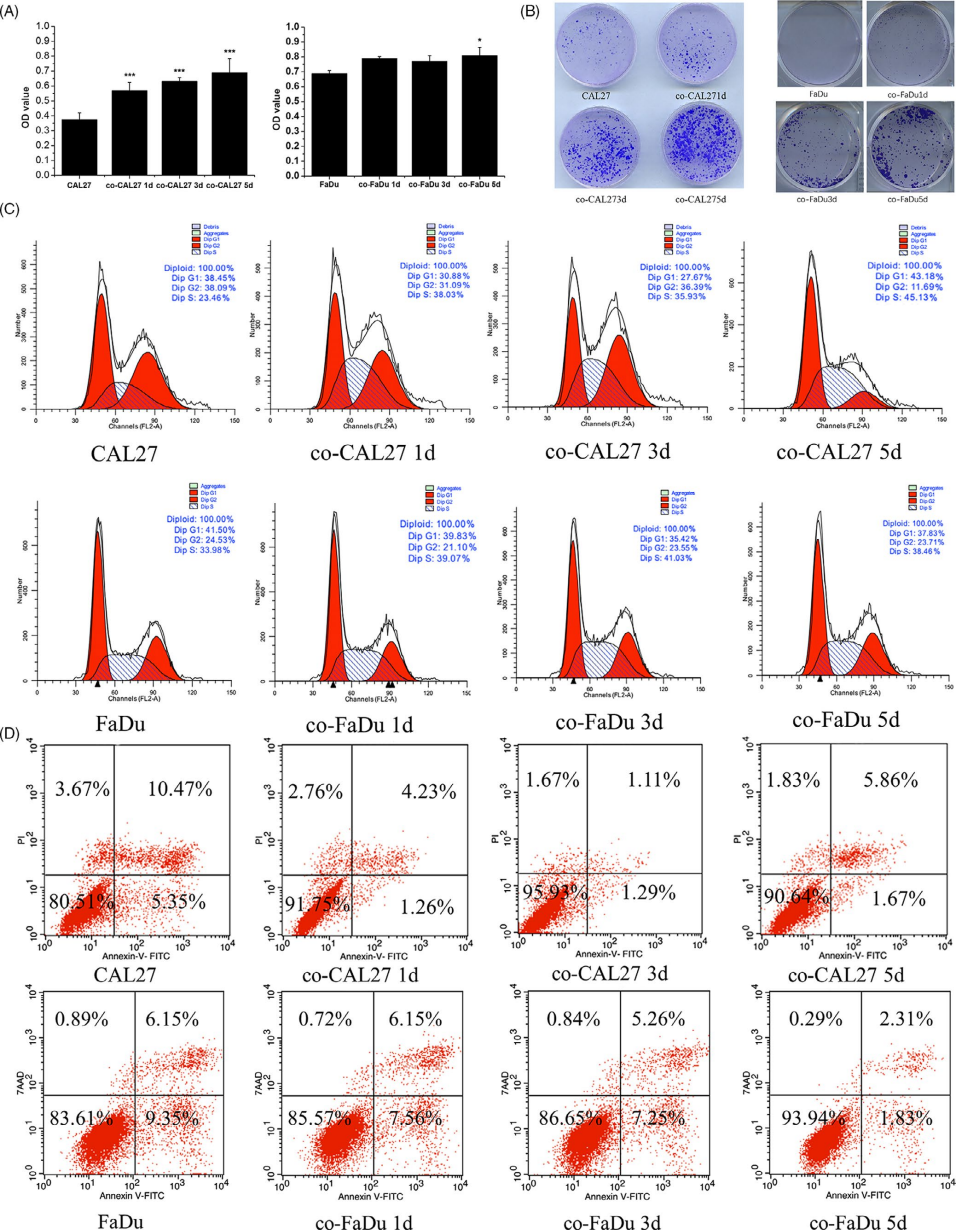
BMSCs promoted proliferation and inhibited apoptosis on OSCC Cell. A, Cell proliferation was assessed using CCK‐8 assay. B, The stemness of OSCC was assessed by colony formation experiment. C, Flow cytometric analysis of cell cycle distribution. D, Annexin V and propidium iodide analysis of OSCC cell apoptosis

Now, we know that the OSCC can induce BMSCs to migrate to OSCC tumour by CXCL8‐CXCR2 interaction, but we still want to know why OSCC needs BMSCs to come near. Therefore, we want to evaluate whether BMSCs affect proliferation and apoptosis of OSCC tumour cells. To explore whether BMSC promoted the proliferation of OSCC cells, CCK‐8 assay was carried out on OSCC cells. After co‐culture with BMSC‐CM at 1 day, 3 days and 5 days, numbers of OSCC cells were significantly increased compared with cells cultured in control media (Figure 3A). The colony formation experiment found that the number of OSCC colony increased after co‐culture with BMSCs, indicating that BMSCs promoted the stemness of OSCC (Figure 3B). Propidium iodide staining was used to determine cell cycle. The results show that BMSC significantly altered the cell cycle distribution of OSCC cells with an increased percentage of cells in the S phase of the cell cycle (Figure 3C). Annexin V and propidium iodide were detected by FACS to determine cell apoptosis. Results showed that the apoptosis rate of OSCC cells co‐cultured with BMSC‐CM was significantly decreased compared with the control group (Figure 3D). These results suggest that BMSC promoted proliferation in OSCC cell and inhibition of cell apoptosis.

### 3.4 BMSC promoted migration of OSCC

**Figure 4:**
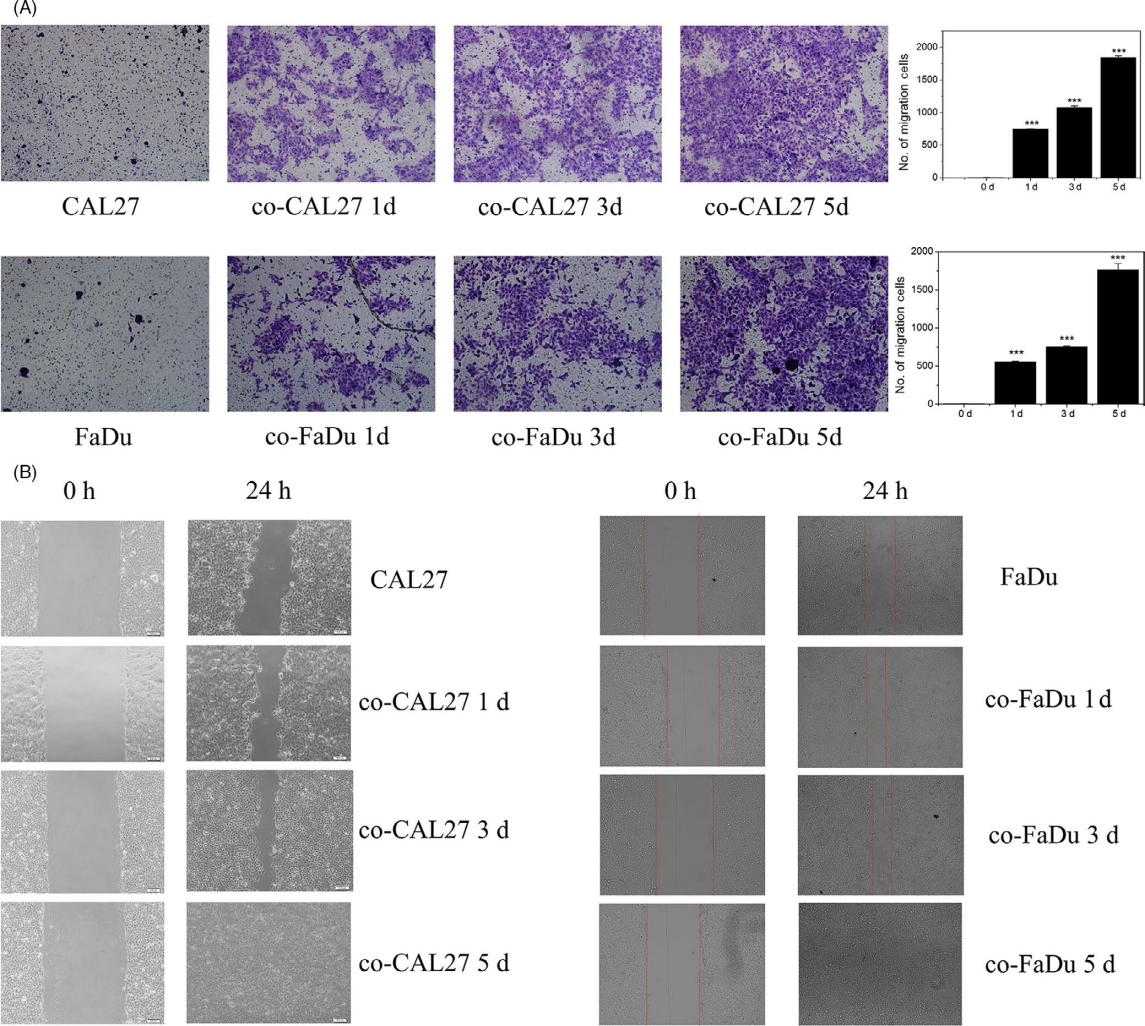
BMSC promoted migration of OSCC. A, Trans‐well invasion assay shows that BMSC promoted the vertical migration of CAL 27 and FaDu cells. B, Scratch assay shows that BMSC promoted the plane migration of CAL 27 and FaDu cells

To identify whether BMSCs promoted CAL27 and FaDu cell migration, we performed a trans‐well invasion assay, and we found that OSCC cells exhibited an enhanced vertical migratory capacity after treatment with BMSC‐CM. Moreover, this migratory capacity increased along with co‐culture time (Figure 4A). As shown in Figure 4B, to further confirm this result, we performed a scratch assay to assess the plane migratory capacity of cells, CAL27 and FaDu cells treated with BMSC‐CM migrated nearly 100% of the scratch within 24 hours, while control groups failed to close the scratch after 24 hours, which produced similar results with trans‐well invasion assay.

### 3.5 BMSCs promoted OSCC cells to express EMT relative molecules through TGF‐β1/Ras/Raf/Erk signalling pathway

**Figure 5:**
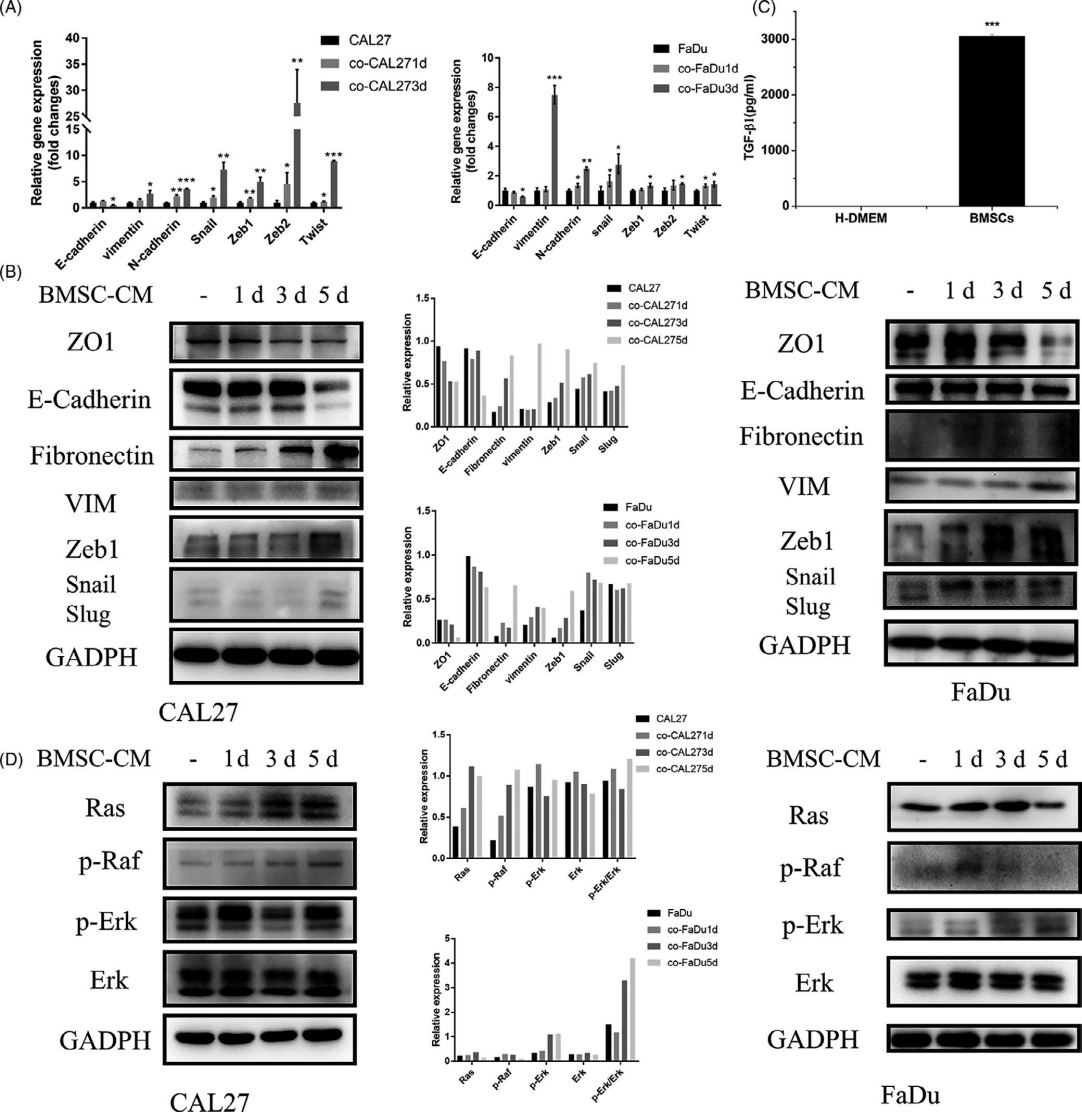
BMSCs promoted OSCC cells that express EMT relative molecular changes by TGF‐β1/Ras/Raf/Erk signalling pathway. A, Real‐time PCR assay detected mRNA expression of E‐cadherin, vimentin, N‐cadherin, snail, zeb1, zeb2 and twist in CAL27 cells. B, Western blot analysis of the expression of E‐cadherin, ZO1, vimentin, fibronectin, snail, slug and Zeb‐1 in OSCC cells in comparison with their control counterparts. C, ELISA detected secretion of TGF‐β1in BMSC‐CM. D, Western blot assay of Ras, p‐Raf and p‐Erk in OSCC cells. *, *P* < .05; **, *P* < .01; and ***, *P* < .001

We have first studied the mRNA expression of epithelial (E‐cadherin) and mesenchymal (vimentin and N‐cadherin) markers and transcriptional factor (snail, zeb1, zeb2 and twist) in different OSCC cell lines with co‐culture (Figure 5A). In OSCC cells, the mRNA expression of E‐cadherin decreased after co‐culture. Meanwhile, mRNA expression of mesenchymal markers showed a significant increased and transcriptional factor. Next, we examined the epithelial (E‐cadherin and ZO‐1), mesenchymal (vimentin and fibronectin), phenotypic and transcriptional factor (snail, slug and zeb1) changes induced by BMSC‐CM in OSCC cells (Figure 5B). We could observe a decrease in the expression of E‐cadherin in co‐culture 5d OSCC cells in comparison with control cells, as well as ZO1. In addition, a significant increase in fibronectin expression was also observed in co‐culture CAL27 cells in comparison with CAL27 cells. However, in co‐culture FaDu cells, the vimentin and fibronectin expressions were not so pronounced. The expression of snail, slug and zeb1 transcriptional factor was also studied (Figure 5B). Results showed an increase in the expression of snail, slug and zeb1 in co‐culture OSCC cells vs their control counterparts. To further investigate the mechanism of how BMSC‐CM promoted the EMT of OSCC cells, cytokines in the BMSC‐CM were detected by ELISA. Figure 5C shows that BMSCs significantly secreted TGF‐β1. In order to search the signalling pathway after TGF‐β1 activation, Ras, phosphorylated Raf and Erk were analysed by Western blot using OSCC cells cultured in control media or BMSC‐CM. Results showed that BMSC‐CM promoted protein expression of Ras, p‐Raf and p‐Erk, which indicated that BMSCs promoted OSCC EMT by activating the TGF‐β1/Ras/Raf/Erk signalling pathway (Figure 5D). TGF‐β1/Ras/Raf/Erk signalling emerged as BMSC‐CM added in OSCC cells. Therefore, we tested whether TGF‐β1/Ras/Raf/Erk regulates EMT activation in OSCC cells. Biological activity of OSCC cells was detected after inhibiting TGF‐β1 and phosphorylated Erk. Results showed that cell proliferation was decreased in CAL27 and FaDu cells (Figure S2A), and migration ability of CAL27 and FaDu was inhibited (Figure S2B) after adding inhibitors. The ICF results showed that expression of E‐cadherin was increased and the expressions of vimentin were decreased in CAL27 and FaDu cells after inhibiting TGF‐β1 and Erk (Figure S2C and Figure S2D). These results suggested that the proliferation ability was inhibited, migration ability was decreased in OSCC cells when TGF‐β1 inhibitor SB431542 or p‐Erk inhibitor U0126 was added to BMSC‐CM, which means that the BMSC‐activated TGF‐β1/Ras/Raf/Erk pathway played important roles in OSCC EMT.

### 3.6 BMSC promoted tumorigenesis and EMT in murine xenograft model of CAL27 cells

**Figure 6:**
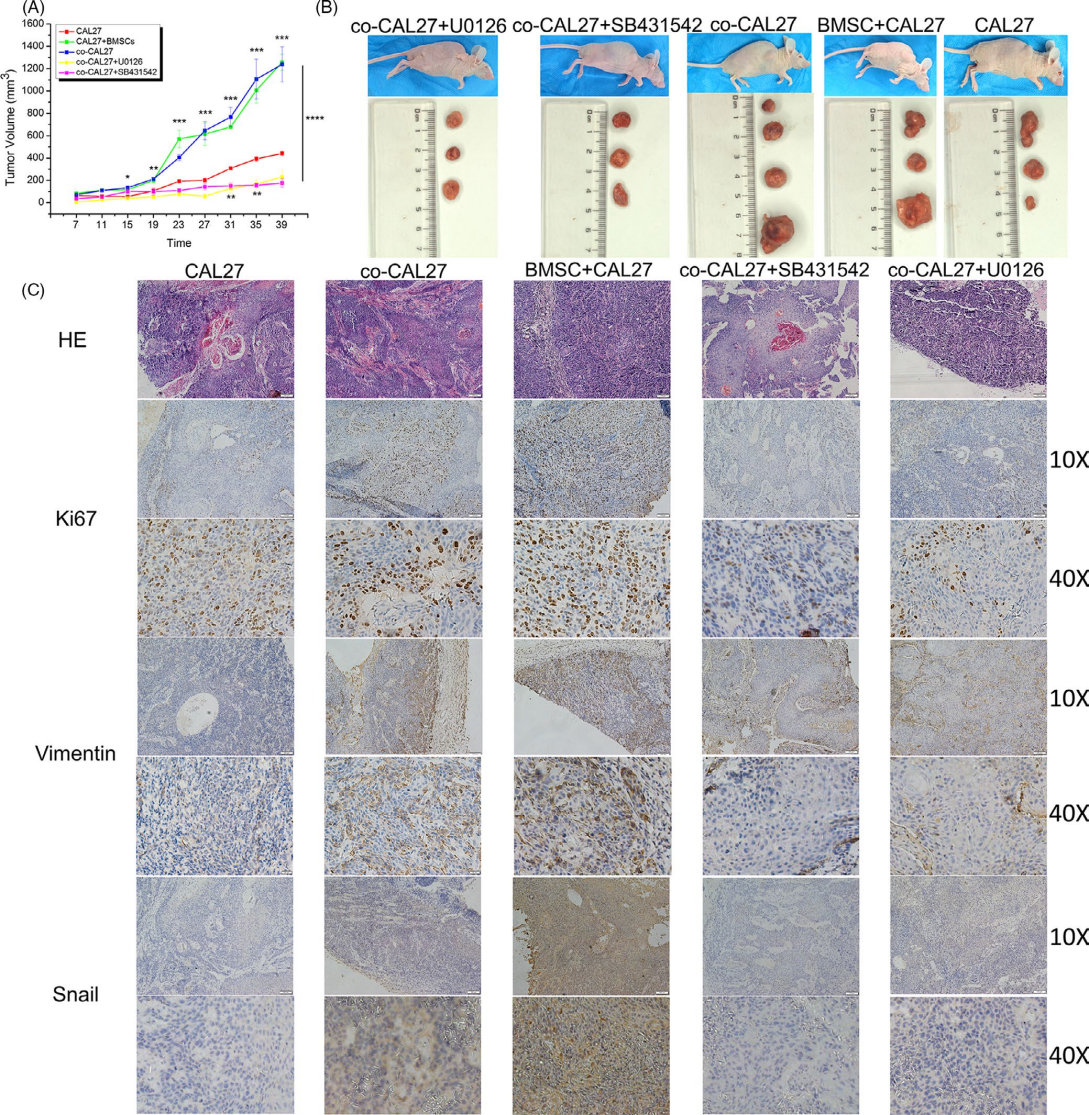
BMSCs promoted tumorigenesis and EMT in murine xenograft model of CAL27 cells. A, The initial in vivo growth analysis of xenografts. There was a significant difference in tumour size between these groups up to day 39 after xenograft implantation in nude mice. B, Representative photographs of xenografts nude mice and xenografts at day 42 after tumour implantation. C, The expression of Ki67, vimentin and snail in xenografts tumours. Representative images of Ki67, vimentin and snail immunohistochemistry at 10× and 40× magnification. *, *P* < .05; **, *P* < .01; and ***, *P* < .001

To explore the effects of BMSCs on OSCC, we performed co‐culture experiments first, in which CAL27 cells were grown with BMSC‐CM and BMSCs for 5 days. Then, we investigated whether BMSCs could affect OSCC tumour growth in vivo. For this purpose, xenograft model of OSCC using CAL27, BMSC‐CM co‐culture CAL27, BMSCs mixed with CAL27, CM co‐culture CAL27 + SB431542 and CM co‐culture CAL27 + U0126 were established in nude mice. During six weeks after injection, tumour volumes were measured every third day. Figure 6A shows that there were significant differences in tumour volume in BMSC‐treated mice compared with control mice; meanwhile, mix culture and CM culture have not difference. Tumours in mice with added inhibitor cells were significantly smaller than those in the control group (Figure 6A and 6B). Furthermore, we observed that the Ki67 of BMSCs + CAL27 and co‐CAL27 groups was strongly positive compared with the control group, but inhibitor groups were lower than the control group. The results suggest BMSCs promote the formation of xenograft tumours. In addition, we found that expressions of vimentin and snail were increased in BMSCs + CAL27 and co‐CAL27 groups compared with the matched CAL27 group. Consistently, the increased vimentin and snail expressions in inhibitor groups were decreased. These results indicate BMSCs also promote the extent of tumour invasion and EMT (Figure 6C).

## 4 DISCUSSION

Up to date, influence of exogenous BMSCs on carcinoma progression remains uncertain. Some studies shown that BMSCs promote carcinoma progression, others show that BMSCs suppress carcinoma progression, and also some studies found BMSCs have no significant impact on carcinoma progression.^7^, ^20^, ^21^, ^22^, ^23^ Our results demonstrate that BMSCs can be recruited into OSCC area and interact with OSCC resulting in promoting the progression of OSCC through the CXCL8/CXCR2 and TGF‐β/Ras/Raf/Erk axes. To fully understand the BMSC biology, we still need to do many experiments to dig deep into exact mechanisms. Our study herein, however, indicates that BMSC is a potential good target to be used to treat or prevent OSCC progression in near future.

Tumorigenesis and tumour growth are caused by multiple genetic mutations under the interactions between genes and environmental factors. Tumours are not only neoplastic cells but also include tumour stromal cells and extracellular matrix components, which interact each other to affect the development and invasion of tumours.^24^ Tumour stromal cells and extracellular matrix components are called as the tumour microenvironment. The TME contains many types of cells, vascular endothelial cells, cancer‐associated fibroblasts, immune cells such as tumour‐associated macrophages, myeloid‐derived suppressor cell, T lymphocytes, B lymphocytes and myoepithelial cells, adipocytes, etc^25^ Cancer cells have bidirectional crosstalk not only with tumour stromal cells and extracellular matrix, but also with recruited and resident cells of mesenchymal cells.^26^ BMSCs possess the ability to migrate towards tumour sites in many kinds of tumours, such as hepatocellular carcinoma.^7^ In our study herein, we found that BMSCs are capable of migration towards OSCC both in vitro and in vivo (Figures 1 and 2). These results have rarely been reported in OSCC. Then, BMSCs respond to OSCC cells in vitro and in vivo by CXCL8‐CXCR2 axis.

CXCL8 or interleukin‐8 belongs to the elastin‐like recombiner (ELR)^+^ CXC chemokine family. CXCL8 is initially produced as a protein of 99 amino acids that undergoes cleavage to form active CXCL8 isoforms, a 77 amino acid peptide in non‐immune cells or a 72 amino acid peptide in monocytes and macrophages. Dimerization of CXCL8 forms the structural basis for receptor binding.^27^ CXCL8 is secreted by different cell types, such as blood monocytes, alveolar macrophages, fibroblasts, endothelial cells and epithelial cells.^28^ Recently, researchers have found that CXCL8 exerts multiple effects on biological activities of tumour cells including proliferation, invasion and migration, which are essential for tumour growth and metastasis, such as breast cancer, lung cancer, prostate cancer and colorectal carcinoma.^29^, ^30^, ^31^, ^32^, ^33^ Interestingly, we also recognized that CXCL8 is positive in human OSCC tissues in situ, and two OSCC cell lines, CAL27 and FaDu, can secrete CXCL8 into CCM in vitro (Figure 1). Also, CXCL8 involves in BMSC migration and induces CXCR2 receptor expression in BMSCs (Figure 1 and Figure S1). Previous studies have reported that CXCL8 can bind to two receptors, CXCR1 and CXCR2. CXCR1 and CXCR2 express on many kinds of cells, leucocytes, keratinocytes, fibroblasts, neurons, endothelial cells, epithelial cells, smooth muscle cells, hepatocytes and melanocytes.^34^, ^35^ CXCR1 and CXCR2 are kinds of G protein–coupled receptors, and activation of receptors can contribute to many actions including angiogenesis and tumour growth. Our results demonstrated that CXCR2 is expressed in BMSCs. Importantly, inhibiting CXCR2 expression can significantly decrease the migration of BMSCs towards OSCC cells in vitro and in vivo (Figure 2), which indicates that the recruitment of BMSCs depends on interaction between CXCL8 and CXCR2 (Figures S1 and S2). These results suggest that CXCL8‐CXCR2 indeed play important roles in tumorigenesis and development of OSCC.

EMT is a critical process of tumorigenesis of epithelial tumours. Upon EMT, cancer cells acquire migratory and invasive properties.^36^ Our data clearly showed that BMSCs promote the EMT of OSCC cells leading to cancer cell proliferation, decrease apoptosis and necrosis of OSCC cells and increase migration of OSCC (Figures 3, 4 and 5).

Further, we have found that TGF‐β/Ras/Raf/Erk signalling pathway has emerged in the OSCC cells after co‐culture (Figure 5 and Figure S2). Recent study has recognized that rapidly accelerated fibrosarcoma (Raf)/mitogen‐activated protein kinase (MEK)/extracellular signal–regulated kinase (ERK) signalling pathway activation plays critical roles in cancer growth, survival and motility, as well as targeted therapy resistance mechanisms during various stages of cancer.^37^ Ras is a membrane‐associated guanine nucleotide–binding protein that is normally activated in response to the binding of extracellular signals, such as growth factors, and acts as a signal switch cycling in Ras/Raf/Erk signalling pathway. On the other hand, Ras can bind to GTP, which forms metabolically active form, rather than to GDP, which forms metabolically inactive form to regulate cell growth.^38^ RAF activation is initiated by Ras‐GTP association with the Ras binding domain (RBD). There are three functional RAF proteins in humans, A‐RAF, B‐RAF and C‐RAF (c‐Raf‐1), which are dependent on activation segment phosphorylation for activity. C‐RAF is an important one, which is the first Raf isoform identified as a potential cellular oncogene.^39^ TGF‐β1 is the most central mediator in the proliferation and EMT of epithelial cells. ERK, a member of the MAPK family, is the major participant in the regulation of cell growth, differentiation and EMT.^40^ In our results, TGF‐β1 and p‐Erk are increased in co‐culture condition, which indicates that both are activated (Figure 4). Importantly, both inhibition assays for TGF‐β1 and p‐Erk result in significantly decreasing the proliferation and migration of OSCC cells in vitro (Figure S2), and significantly decreasing tumour size compared with the control group in vivo (Figure 6). These suggest that the TGF‐β/Ras/Raf/Erk signalling pathway is involved in the EMT of OSCC stimulated by BMSCs.

Our data demonstrate that OSCC, OSCC of EMT and BMSCs have complicate interactions between them during OSCC development and progression. They can recruit and promote each other through CXCL8 secreted by OSCC and CXCR2 expressed in BMSCs interactions leading to activate TGF‐β/Ras/Raf/Erk signalling pathway resulting in proliferation, invasion and migration of OSCC. BMSC is a critical component of the host‐response network in tumours and provides a potential novel anti‐cancer target for the treatment of OSCC. Data from the in vivo experiments (Figure 6) may also suggest that the time we used for co‐culture with BMSCs or condition medium was enough to reprogramme CAL27 cells or turn on some important signals in the CAL27 cells resulting in CAL27 cell progressive growth. This is our new hypothesis needed to further study. In this study, we still do not understand effects of BMSCs on normal epithelial cell, such as HaCaT from our current study although we demonstrate that BMSCs can promote tumour. We will perform more in vitro and in vivo experiments to reveal the truth in the further study.

## CONFLICT OF INTEREST

The authors declare that they have no competing interests.

## AUTHOR CONTRIBUTIONS

ML contributed to conception and design, data acquisition, analysis and interpretation, drafted and critically revised the manuscript; ZY contributed to data acquisition, analysis and interpretation, and critically revised the manuscript; BW contributed to design and data acquisition, and critically revised the manuscript; LX contributed to design and data acquisition, and critically revised the manuscript; LX, ZD, CY and ZS contributed to data acquisition and critically revised the manuscript; LQ contributed to design and critically revised the manuscript; SH and L.QL. contributed to conception, design, data acquisition, analysis and interpretation, and drafted and critically revised the manuscript. All authors gave final approval and agreed to be accountable for all aspects of the work.

## Supporting information

Supplementary Material

Fig S1

Fig S2

Fig S3

Table S1

## Data Availability

The data that support the findings of this study are available within the article and its Appendix or on reasonable request from the corresponding author.
