# Supplementary material for: Bone mesenchymal stem cells are recruited via CXCL8‐CXCR2 and promote EMT through TGF‐β signal pathways in oral squamous carcinoma
Source: Cell Prolif. 2020 Jun 26;53(8):e12859. doi: 10.1111/cpr.12859 (PMC7445409; doi:10.1111/cpr.12859)
Supplement: Supplementary file 1 — Supplementary Material [file CPR-53-e12859-s001.docx]

**Supplementary Information**

1. MATERIALS AND METHODS

**Cell cultures**

Human OSCC cell lines, CAL27, FaDu and human skin cell line, Hacat were obtained from Nanjing Keygen Biotech Company (China). All cells grew in DMEM (Gibco) containing 10% FBS (Biological Industries) with 100 units/ml penicillin and 100 μg/ml streptomycin (HyClone) in a 37°C humidified incubator containing 5% CO2. BMSCs were treated with 10 nM inhibiting CXCL8 binding to CXCR2 SB225002 (Selleck) for 24 hours. CAL27 and FaDu were treated with 10 nM TGF-β inhibitor SB431542 (Selleck) and ERK inhibitor U0126 (Med Chem Express) respectively.

**Immunohistochemistry**

The paraffin-embedded tissues sections (3.5 μm) on glass slides were deparaffinized and hydrated, and submerged into EDTA buffer for heat-induced antigenic retrieval (pH 8.0, Zsbio). The intrinsic peroxidase activity and nonspecific antibody binding were blocked using Ultra Sensitive TM SP kit (Maxim). Primary antibodies were used at the following dilutions, CXCL8 (1:200, Santa cruz), ki67 (1:2000, Proteintech), vimentin (1:1000, Proteintech), snail (1:1000, Proteintech) overnight at 4°C. Slides were developed using the UltraSensitive TM SP kit and DAB kit (Maxim), followed by coun-terstaining with hematoxylin, dehydration, clearing and mounting with neutral gums.

**Immunofluorescence histochemical double staining**

Paraffin-embedded tissues sections (3.0 μm) were deparaffinized and hydrated, and submerged into EDTA buffer for heat-induced antigenic retrieval (pH 8.0, Zsbio). The intrinsic peroxidase activity and nonspecific antibody binding were blocked using 10% BSA-PBS. Slides were incubated with CXCL8 (1:200, Santa cruz) overnight at 4 °C, washed, incubated 2 h at 37°C with Cy3–conjugated IgG (1:50, Proteintech), and then were incubated with CD105 (1:1000, Proteintech), washed, incubated by FITC–conjugated IgG (1:50, Proteintech) and co-stained 5 min with 4,6-diamidino-2-phenylindole to visualize cell nuclei. Images were obtained using a fluorescence microscope.

**Cell immunofluorescence**

Cells were seeded on the clean glass cover slips and cultured overnight. Medium was aspirated, cells were briefly washed three times with PBS, fixed with fresh 4% paraformaldehyde in PBS for 10 min, rinsed with PBS 3 times for 3 min/each, permeabilized with 0.2% Triton X-100 for 5 min, again rinse 3 times with PBS for 3 min/each, incubated with 5% BSA-PBS of block solution for 1 hour, aspirated blocking solution and incubated with primary antibody overnight at 4°C, then washed 3 times with PBS for 3 min/each, and incubated with fluorescence-labeled secondary antibody (Proteintech) for 1 h at room temperature and counterstained with DAPI for 5 min. Primary antibodies were used at the following dilutions: E-cadherin (1:1000 dilution, Proteintech), vimentin (1:1000 dilution, Proteintech).

**Collection of conditioned medium**

80% confluence of CAL27, FaDu, Hacat and BMSC were cultured in 10 ml serum-free medium for 24 h, and each supernatant was collected as cell conditioned medium (CCM). All CCMs were centrifuged by 1500 r/min to scavenge cell debris and then stored at -70℃ until used.

**In Vitro Migration Assay**

Ability of BMSC to migrate toward OSCC cells was measured using trans-well chambers with 8 μm filter membranes (Corning). CCM collected from CAL27, FaDu and Hacat were added in bottom chamber. For blocking experiments, CCM were added in CXCL8-CXCR2 inhibitor. We also assessed the migration of BMSCs toward increasing concentrations of human recombinant CXCL8(AF-200-08M, Peprotech). In all migration experiments, BMSCs were suspended in serum-free medium, and migration in response to the same medium served as a negative control. After 24 h, BMSCs were fixed by 4% paraformaldehyde, and stained by crystal violet. Then migrated cells were quantified in five view fields on each membrane, and all experiments were repeated in triplicate.

**Reverse Transcriptase Quantitative Polymerase Chain Reaction (RT-qPCR) assay**

To detect gene expression, RNA was extracted using Trizol reagent (Invitrogen). cDNAs were synthesized using PrimeScript 1st strand cDNA Synthesis Kit (Takara Bio). Primers (table) were used to evaluated different gene expressions. β-actin were used as internal control. The qPCR assays were performed using SYBR Green Premix Ex Taq (Takara Bio) and MxPro M ×3005P real-time PCR detection system (Agilent Technologies). All experiments were repeated in triplicate.

Table S1 Primers used in this study

**ELISA Detection of Human Chemokine CXCL8**

Concentrations of CXCL8 in the CCM of OSCC cells were determined using Human IL-8 (CXCL8) ELISA development kit (HRP) (MabTech, Nacka Strand, Sweden). Concentrations of TGF-β1 in the CCM of BMSCs were determined using Human TGF-β1 Precoated ELISA kit (DAKEWE, Bei Jing, China).

**Western blot**

Cells were washed with PBS and lysed in RIPA buffer containing protease inhibitor (Med Chem Express) and phosphatase inhibitor (Med Chem Express). Protein samples were electrophoresed through 8-12% SDS-PAGE. After transferring proteins from gel to PVDF membrane, membrane was blocked with 5% BSA in TBST and incubated with primary antibodies overnight at 4°C, then washed and incubated with secondary antibodies (Proteintech). Membrane was detected using enhanced chemiluminescence rea-gent (Proteintech). Results were normalized by internal control, GAPDH.

Primary antibodies, ZO1(1:500 dilution, Proteintech), E-cadherin (1:1000 dilution, Proteintech), Fibronectin (1:500 dilution, Proteintech), vimentin (1:1000 dilution, Proteintech), Zeb1(1:500 dilution, Santa cruz), Snail+Slug (1:1000 dilution, Abcam, Cambridge, MA, USA), Ras (1:500 dilution, Santa cruz), p-Raf 1:1000 dilution, Cell Signaling Technology, Danvers, MA, United States), p-Erk (1:1000 dilution, Cell Signaling Technology), Erk (1:1000 dilution, Cell Signaling Technology) were used in this study.

**Particle synthesis and characteristics of Au-PEI**

Au-PEI is one of gold nanoclusters. Prepared PEI-SH ligand (0.3 mL, 3 mM) was mixed with 5 mL deionized water, following with adding HAuCl_4_ aqueous solution (0.25 mL, 50 mM). Then, 0.3 mL N_2_H_4_H_2_O was dropped into the solution. The mixture was finally reacted for 4 h at 80 °C with vigorous stirring. The color of the solution changed from light yellow to brown, which were Au-PEI. Au-PEI were washed twice with acetone and dialyzed (MW cutoff = 3,500 Da) for 48 h.

**Cell imaging**

BMSCs were cultured at 5 x 10^4^ cells/well on the Nunc Glass Bottom Dish overnight. Then, medium was replaced with FBS-free H-DMEM containing 40ium was replaced with FBS-free H-DMEM containing 40or of the solution changed from light yellow to b℃ for 30 min, and washed with PBS. All samples were examined using a confocal laser scanning microscope (CLSM) and inverted fluorescence microscope with an excitation of 420 nm.

**In Vitro Effect of BMSCs on OSCCs**

To evaluate effects of BMSCs on OSCCs, four groups were set up, OSCCs cultured with H-DMEM during 5 days, OSCCs cultured with BMSC-CM on the fourth day; OSCCs cultured with BMSC-CM on the second day; OSCCs cultured with BMSC-CM during 5 days.

**Cell Proliferation Assay**

OSCC cells were cultured at 1 x 10^4^ cells/well in 96-well plates for 24 h. Six duplicates were for each group. After 24 h, 10μl of CCK-8 (Vazyme, Piscataway, NJ, USA) solution was added to each well and incubated for 1.5 h at 37°C. OD value was detected with a microplate reader at a wavelength of 450 nm.

**Cell Cycle Assay**

Cells were cultured at 2 x 10^5^ cells/well in 6-well plates for 24 h. Cells were resuspended in PBS, washed once then were incubated in 10 μl of Permeabilization solution and 1ml of DNA Staining solution of Cell Cycle Staining Kit (MultiSciences, Hung Zhou, China). BD FACS Aria II SORP (BD biosciences, Mississauga, Canada) was used for FACS. For each experiment, 10,000 events were counted, and cell cycle profiles were modeled using Modfit software (Verity Software House, Topsham, ME, USA).

**Cell Apoptosis Assay**

Cells were seeded at 2 x 10^5^ cells/well in 6-well plates for 24 h. Then, apoptotic cells were detected using FITC Annexin V and propidium iodide (BD Biosciences, Sparks, MD, USA). Briefly, after cells were washed with cold PBS, cells were resuspended in 100 μl of binding buffer with 5 μl FITC-Annexin V and propidium iodide and then incubated for 15 min at room temperature. Numbers of apoptotic cells were determined by flow cytometry.

**Colony Formation Assays**

For colony formation assays, cells were cultured 500 cells/well in 6-well plates for 20 days. Triplicate was for each group. Medium was replaced every two weeks. Cells were fixed in 4% paraformaldehyde, stained with 0.5% crystal violet and observed under microscope.

**Trans-well Infiltration Assay**

Cells were cultured at 1 x 10^4^ cells/well in the insert/trans-well (Corning Inc., Corning, NY, USA) for 6-well plates with 200 μl serum-free H-DMEM in trans-well chamber and 600 μl H-DMEM in the lower chamber for 24 h. After 24 h, the membranes were fixed with 4% paraformaldehyde and stained with crystal violet solution. Herein trans-well chambers were coated by matrigel. Cells on the upper surface of the membrane were removed and cells that had migrated through the membrane were imaged under a light microscope and quantified using Image J software. All experiments were repeated in triplicate and 5 images were took per membrane.

**Wound-healing Assay**

Cells were cultured at 3 x 10^5^ cells/well in 6-well plates in triplicate wells. plates were scraped with a 200 μl pipette tip, and washed with cell culture media 3 times. Then, cells were incubated with se-rum-free medium. Wounded areas were photographed at 0, 6, 12 and 24 h under a microscope. Size of the wound area and closure of the wound were analyzed.

**2. SUPPLEMENTAL FIGURES**

**Figure S1.** CXCR2 mediates the migration of BMSCs to OSCC In Vitro. (A) Re-al-time polymerase chain reaction analysis of mRNA expression of CXCR1 and CXCR2 in BMSCs after co-culture CAL27. CXCR2 mRNA was detected in BMSCs (B) mRNA expression of CXCR1 and CXCR2 in BMSCs after co-culture FaDu. CXCR2 was positive, but BMSCs were negative for CXCR1. (C) Inhibition of CXCR2 on BMSCs significantly inhibited the migration of BMSCs toward CCM from OSCC. (D) Migration of BMSCs toward CCM from OSCC cell lines added inhibitor. ***, p < 0.001.

**Figure S2.** Regulation of EMT via TGF-β1/Ras/Raf/Erk signaling pathway in OSCC cells (A) Cell proliferation ability detected by CCK-8 assay. (B) Cell migration ability detected by Trans-well assay in OSCC cells. (C, D) E-cadherin and vimentin expression are detected by ICF. E-cadherin expression shown in red, and vimentin expression shown in green, the nuclei are stained by DAPI (blue) (×10). *P < 0.05, **P <0.01, ***P<0.001.

**Figure S3.** Morphology and surface marks of BMSCs. (A) CD90, CD105, CD44, CD45 and CD11b analyses by flow cytometry. (B) Cell morphology of BMSCs from second passage.
