# Supplementary figures and images for: Bone mesenchymal stem cells are recruited via CXCL8‐CXCR2 and promote EMT through TGF‐β signal pathways in oral squamous carcinoma
Source: Cell Prolif. 2020 Jun 26;53(8):e12859. doi: 10.1111/cpr.12859 (PMC7445409; doi:10.1111/cpr.12859)

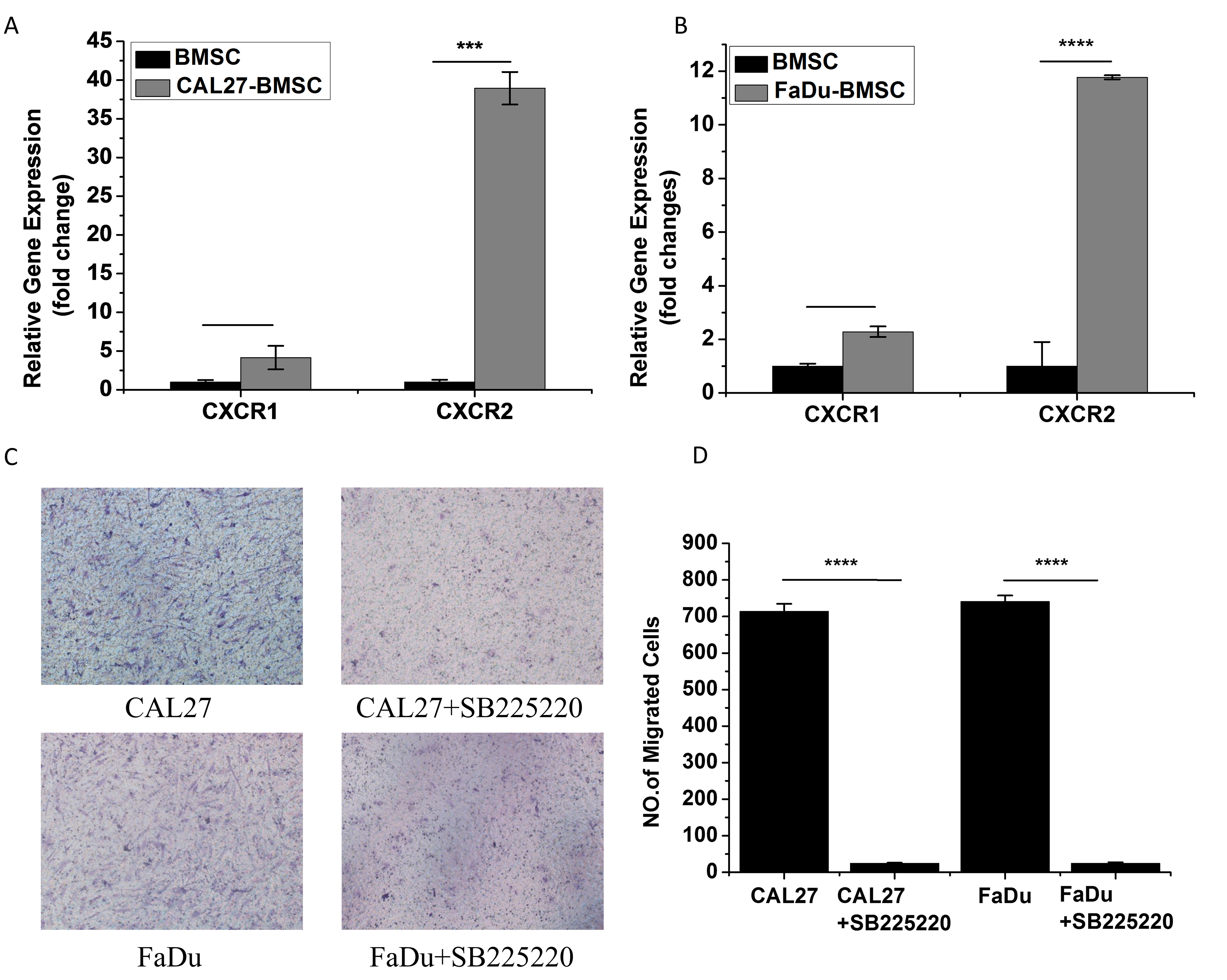

Supplement: Supplementary file 2 — Fig S1 [file CPR-53-e12859-s002.tif]

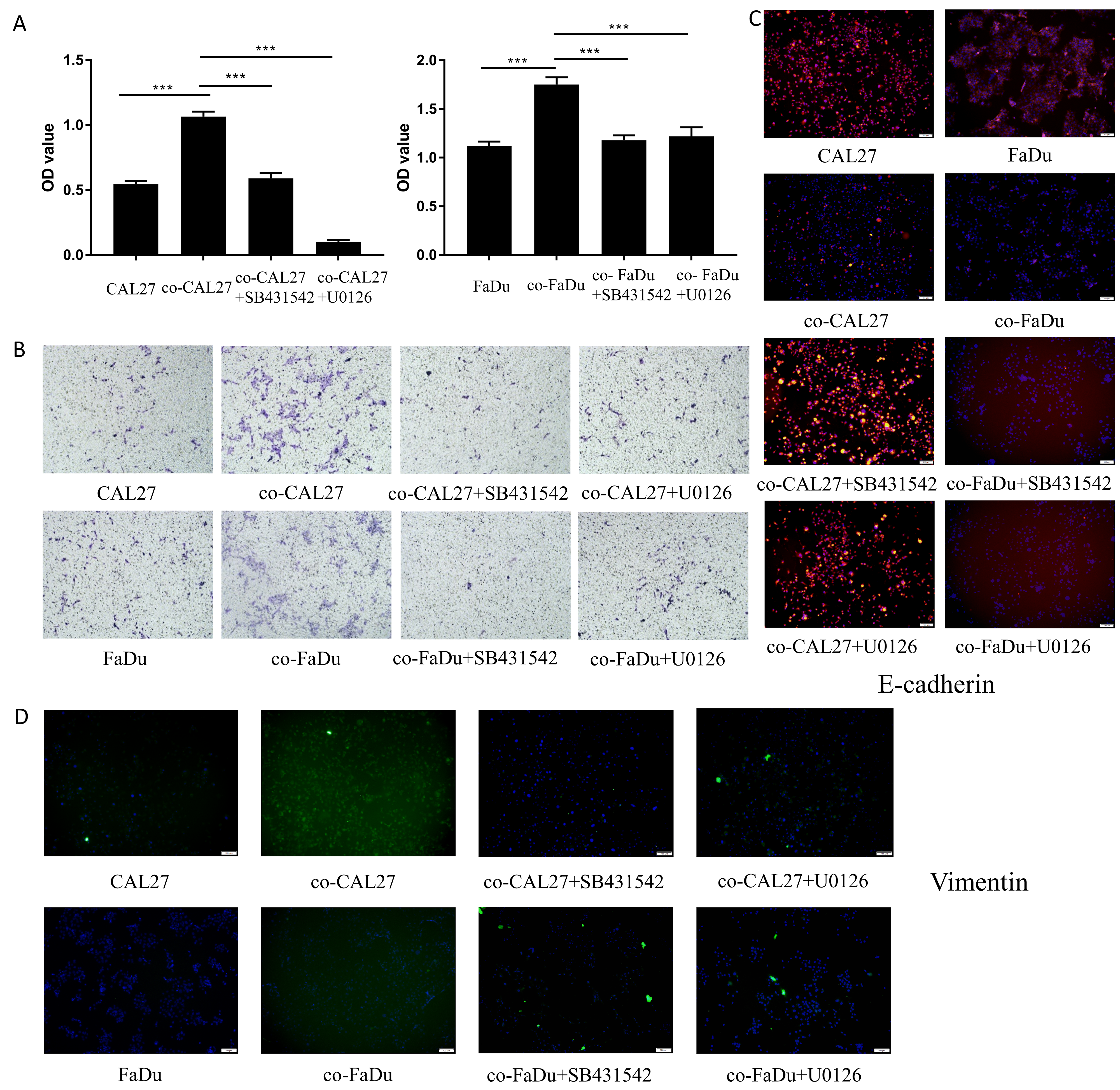

Supplement: Supplementary file 3 — Fig S2 [file CPR-53-e12859-s003.tif]

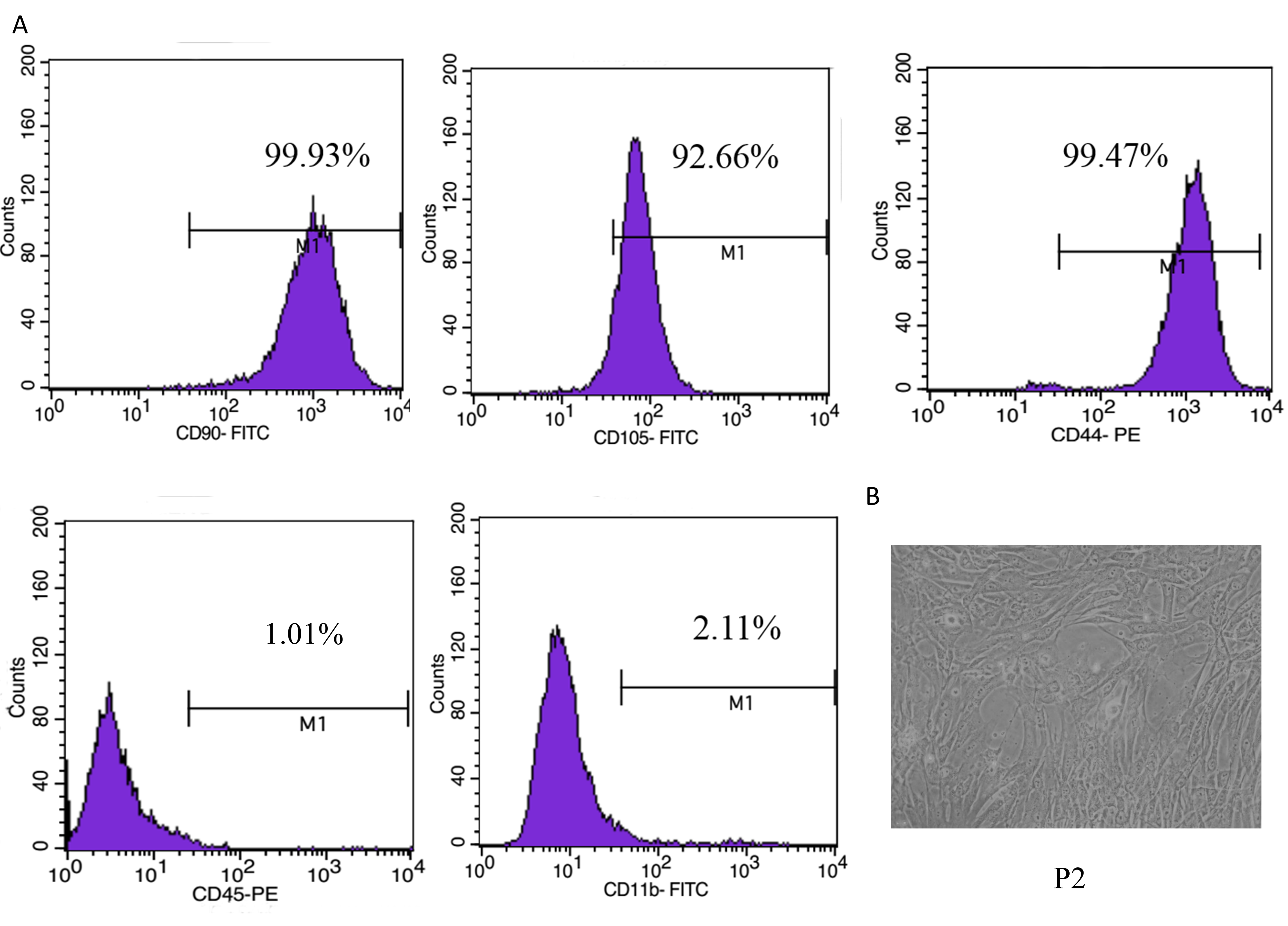

Supplement: Supplementary file 4 — Fig S3 [file CPR-53-e12859-s004.tif]
