# Supplementary material for: Bone mesenchymal stem cells are recruited via CXCL8‐CXCR2 and promote EMT through TGF‐β signal pathways in oral squamous carcinoma
Source: Cell Prolif. 2020 Jun 26;53(8):e12859. doi: 10.1111/cpr.12859 (PMC7445409; doi:10.1111/cpr.12859)
Supplement: Supplementary file 5 — Table S1 [file CPR-53-e12859-s005.docx]

Table S1 Primers used in this study

| Genes | Forward primers | Reverse primers |
| --- | --- | --- |
| CCL2 | 5′- CTTCTGTGCCTGCTGCTCATA -3′ | 5′- CTTTGGGACACTTGCTGCTG -3′ |
| CCL3 | 5′- CCTGCTCAGAATCATGCAGGTC -3′ | 5′- CACTGGCTGCTCGTCTCAAAG -3′ |
| CCL5 | 5′- TCGCTGTCATCCTCATTGCTA -3′ | 5′- GGAGCACTTGCCACTGGTGTA -3′ |
| CXCL16 | 5′- TCCCACAGCCAGGACATCAG -3′ | 5′- TCAGGTATTAGAGTCAGGTGCCACA -3′ |
| CXCL8 | 5′- ACACTGCGCCAACACAGAAATTA -3′ | 5′- TTTGCTTGAAGTTTCACTGGCATC -3′ |
| CXCL1 | 5′-CAAACCGAAGTCATAGCCACAC -3′ | 5′- GGATTTGTCACTGTTCAGCATCTT -3′ |
| CXCR1 | 5′- TGTTAAGCGTTGAGCCACCAAG -3′ | 5′- TCTCCAGCAGACACTGCAACA -3′ |
| CXCR2 | 5′- TCTTCAGGGCACACTTCCACTAC -3′ | 5′- GGGCTGCATTGACACTGAGA -3′ |
| E-cadherin | 5′- GAGTGCCAACTGGACCATTCAGTA -3′ | 5′- AGTCACCCACCTCTAAGGCCATC -3′ |
| Vimentin | 5′- AACCTGGCCGAGGACATCA -3′ | 5′- TCAAGGTCAAGACGTGCCAGA -3′ |
| N-cadherin | 5′- CTGCAGCAGCCTGACACTGT -3′ | 5′- GCAGATCGGACCGGATACTG -3′ |
| Snail | 5′- CTGTGACAAGGAATATGTGAGC -3′ | 5′- CTAATGTGTCCTTGAAGCAACC -3′ |
| Zeb1 | 5′- CAGGCAAAGTAAATATCCCTGC -3′ | 5′- GGTAAAACTGGGGAGTTAGTCA -3′ |
| Zeb2 | 5′- GAAGACAGAGAGTGGCATGTAT -3′ | 5′- GTGTGTTCGTATTTATGTCGCA -3′ |
| Twist | 5′- GTACATCGACTTCCTCTACCAG -3′ | 5′- CATCCTCCAGACCGAGAAG -3′ |
| β-actin | 5′-TGGCACCCAGCACAATGAA-3′ | 5′-CTAAGTCATAGTCCGCCTAGAAGCA-3′ |
